# Evaluation of antioxidant, anti-inflammatory, anticancer activities and molecular docking of *Moringa oleifera* seed oil extract against experimental model of Ehrlich ascites carcinoma in Swiss female albino mice

**DOI:** 10.1186/s12906-023-04279-z

**Published:** 2023-12-14

**Authors:** Tahany Saleh Aldayel, Heba N. Gad El Hak, Mohamed S. Nafie, Raneem Saad, Heba M. A. Abdelrazek, Omnia E. Kilany

**Affiliations:** 1 Department of Health Sciences, Clinical Nutrition, College of Health and Rehabilitation Sciences, Princess Nourah bint Abdulrahman University, Riyadh, 11671 Saudi Arabia; 2 Department of Zoology, Faculty of Science, Suez Canal University, Ismailia, Egypt; 3 Chemistry Department, Faculty of Science, Suez Canal University, Ismailia, 41522 Egypt; 4 Department of Clinical Pathology, Faculty of Veterinary Medicine, Suez Canal University, Ismailia, Egypt; 5 Department of Physiology, Faculty of Veterinary Medicine, Suez Canal University, Ismailia, Egypt

**Keywords:** Antioxidants, Cytokines, Ehrlich ascites carcinoma, Liver, Kidney, *Moringa olifera* oil extract

## Abstract

The current research intended to evaluate the antitumor properties of *Moringa oleifera* oil extract (MOE). Fifty-six female Swiss albino mice were employed in this study. Animals were assigned into four groups: control (C) group, moringa oil extract (MOE) group administered (500 mg/kg b. wt) MOE daily via gavage, Ehrlich ascites carcinoma (EAC) group and EAC group administered daily with (500 mg/kg b.wt) MOE for two weeks (EAC/MOE). The results showed that MOE significantly ameliorated the EAC increase in body weight and reduced the EAC cell viability. In addition, they upgraded the levels of hepatic and renal functions, inflammatory cytokines, oxidative stress markers and EAC-induced hepatic and renal histopathological changes. Treatment of EAC with MOE induced antitumor, anti-inflammatory and antioxidant effects and normalized most of the tested parameters besides the histopathological alterations in both renal and hepatic tissues. HPLC for the MOE identified Cinnamic acid, Ellagic acid, Quercetin, Gallic acid, Vanillin and Hesperidin as major compounds. The molecular docking study highlighted the virtual binding of the identified compounds inside the GSH and SOD proteins, especially for Quercetin which exhibited promising binding affinity with good interactive binding mode with the key amino acids. These results demonstrate that the antitumor constituents of MOE against EAC induced oxidative stress and inflammation by preventing oxidative damage and controlling EAC increase.

**Supplementary Information:**

The online version contains supplementary material available at 10.1186/s12906-023-04279-z.

## Introduction

Cancer is a death leading cause worldwide [1]. Despite years of pre-clinical and clinical research, as well as trials of promising new therapies, mortality due to cancer prevalence continues to increase [2]. Oxidative stress is a pivotal contributor to the pathogenesis of several illnesses [3]. Moreover, oxidative stress causes many alterations in both the function and structure of the cell in addition to mutagenesis in deoxyribonucleic acid, leading to the development of cancer [4]. Numerous cytokines are produced in the tumor microenvironment and play an essential role in cancer pathogenesis [5]. In addition, numerous cytokines are released in response to inflammation and immunity to inhibit tumor development and progression [6].

Animal models of cancer have been employed to simulate human natural tumors [7], enabling us to study the machinery of tumor progression as well as its behavior [8]. In addition, the selection of an animal model is crucial for obtaining an accurate exponent during research on cancer progression [9]. Ehrlich ascites cancer (EAC) is one of the most common experimental tumor models [10]. EAC are known as undifferentiated cancers that have high transplant ability, no regression, rapid growth, short lifespan and 100% tumor malignancy with no tumor-specific transplantation antigen [11]. EAC are parallel to human tumors in terms of sensitivity to chemotherapy, are undifferentiated and have an accelerated growth rate [12].

The use of alternative cancer therapies, such as natural plants and their products, is believed to have a large contribution in the destruction of cancer and its control [13]. It has been estimated that 60% of the approved drugs used for treating cancer are derived from natural sources [14]. Various medicinal plants have been used to treat and prevent several cancers [15]. *Moringa oleifera* is an important tropical plant that can be used in human nutrition, medicine, and industrial production [16]. Every part of this plant is practically used for the treatment of several human diseases and health concerns [17]. The biological properties of this plant include antioxidant, hepatoprotective [18], hypocholesterolemic [19], hypolipidemic, anti-atherosclerotic [20], immune enhancer [21], wound healing, antidiabetic [22] and antitumor effects [23].

Several researchers have investigated the in vitro effect of MOE on cancer [24]. The study by S Sreelatha, A Jeyachitra and P Padma [25] demonstrated that *Moringa oleifera* leaf extract could be a potential chemopreventive agent. The presence of fatty acids [26] and glucosinolate [27] could have been attributed to the chemopreventive effect of *Moringa oleifera* extract, which modulates apoptosis and inhibits the growth of human cancer cell lines. In addition, the presence of niazimicin and glucomoringin which have been reported to inhibit tumor cell proliferation were also mentioned as possible compounds contributing to the anti-colon carcinogenic effects of *Moringa oleifera* extract [28]. AK Al-Asmari, SM Albalawi, MT Athar, AQ Khan, H Al-Shahrani and M Islam [29] have explored the influences of *Moringa oleifera* bark and leaves on colorectal HCT-8 and breast MDA-MB-231 cancer cells. They demonstrated that *Moringa oleifera* extracts induced marked changes in the cell phenotypic properties of both cell lines. Also, they induced apoptosis-mediated cell death and cell cycle arrest. Moreover, the analyses of these extracts revealed substantial ingredients with anti-cancer prosperities [29, 30]. Parallel et al., [31] evaluated the anti-cancer influence of the Moringa leaf and bark extracts on hepatic cancer cell line (HepG2) using MTT assay. They demonstrated a superior anticancer effect of the leaf crude extract on HepG2 cells than the bark extract of Moringa. Another study revealed that *Moringa oleifera* seed oil extract (MOE) contains several bioactive compounds with antitumor activity [32]. Niazimicin, a bioactive compound found in MOE, exhibits anticancer activity [33]. MOE contains a several compounds with high antioxidant activities [34]. There is scarcity of research and reports demonstrating the antitumor effects of MOE in vivo or against Ehrlich ascites carcinoma animal model. Therefore, the herein study was directed to assess the effectiveness of MOE to diminish EAC, mitigate inflammation, oxidative damage and complications induced by EAC in mice.

## Results

### Body weight

At week 1, individual body weight showed a significant (*P* ≤ 0.05) increase in the EAC inoculated mice in comparison to the control group. The administration of MOE to EAC mice diminished (*P* ≤ 0.05) the body weight in comparison to the untreated EAC mice. Non-significant differences were noted between the MOE and control groups. At week 2, the body weight was (*P* ≤ 0.05) higher in the EAC inoculated mice than those of the control. Treatment of EAC mice with MOE decreased (*P* ≤ 0.05) the body weight to a comparable level to the normal control. Both control and MOE groups did not significantly differ (Table 1).

**Table 1:** Effect of *Moringa* seed oil extract on body weight (g) of EAC mice

Groups	C	MOE	EAC	EAC/MOE
Duration				
Week 1	23.33 ± 0.90^b^	23.33 ± 0.90^b^	29.0 ± 1.50^a^	26.33 ± 0.90^b^
Week 2	24.00 ± 0.60^cd^	24.00 ± 0.60^cd^	30.70 ± 0.90^a^	26.66 ± 0.90^bc^

Values are presented as means (*n* = 14 mice/group) ± SE; different letter superscripts in the same row considered significant at (*P* ≤ 0.05)

*C* Control, *MOE Moringa* seed oil extract, *EAC* Ehrlich ascites carcinoma, *EAC/MOE* Ehrlich ascites carcinoma treated with *Moringa olifera* oil extract

### Viability (%) of EAC cells

Table 2 showed the EAC cell viability at weeks 1 and 2 after MOE administration. At week 1, treatment of EAC mice with MOE produced a significant (*P* ≤ 0.05) decline in the tumor cells’ viability within ascitic fluid when matched to EAC mice. At week 2, the treatment of EAC mice with MOE produced a significant (*P* ≤ 0.05) decline in EAC viability compared to Ehrlich that received no treatment mice.

**Table 2:** Effect of *Moringa* seed oil extract on viability (%) of mice EAC cells

Groups	EAC	MOE
Duration		
Viability at week 1	71.80 ± 3.10^a^	60.40 ± 1.90^b^
Viability at week 2	69.30 ± 0.20^a^	26.90 ± 2.70^c^

Values are presented as means (*n* = 14 mice/group) ± SE; different letter superscripts in the same row considered significant at (*P* ≤ 0.05)

*C* Control, *MOE Moringa* seed oil extract, *EAC* Ehrlich ascites carcinoma, *EAC/MOE* Ehrlich ascites carcinoma treated with *Moringa olifera* oil extract

### Hematology

Table 3 demonstrated the hematological parameters at weeks 1 and 2 after MOE administration in EAC mice. The RBC count, Hb value and PCV% showed a drop (*P* ≤ 0.05) in the EAC mice as compared to the control ones in the first week. Administration of MOE to EAC mice significantly (*P* ≤ 0.05) promoted Hb content, RBC count and PCV% as compared to the non-treated EAC group. Blood indices (MCH, MCV and MCHC) exhibited non-significant (*P >* 0.05) variations for the two control groups (C and MOE). However, these indices showed a significant decline (*P* ≤ 0.05) in the non-treated EAC group when compared to the control. Treatment of EAC with MOE significantly promoted (*P* ≤ 0.05) MCV, MCH and MCHC in comparison to the untreated group. Treatment with MOE in EAC mice revealed a significant (*P* ≤ 0.05) amelioration in the increased WBC count when matched to the non-treated EAC group. At week 2, the RBC count, Hb value and PCV% were not significantly different in the control MOE group compared to the control one (*P* < 0.05). Meanwhile, these parameters were lower (*P* ≤ 0.05) in the EAC mice than the control ones. Administration of MOE to EAC mice increased (*P* ≤ 0.05) the RBC count, Hb content and PCV% to a level similar to that of the control. The blood indices (MCV, MCHC and MCH) did not exhibit alterations (*P > *0.05) in the C and MOE groups. However, these indices showed a significant *(P* ≤ 0.05) reduction in the non-treated EAC group as compared to the control group. Treatment with EAC and MOE enhanced (*P* ≤ 0.05) MCV, MCH and MCHC to a level close to that of the control. In addition, the WBC exhibited non-significant alterations (*P >* 0.05) in the C and MOE groups. Meanwhile, an increase was observed (*P* ≤ 0.05) in the EAC non-treated mice in comparison to the control. Treatment of EAC mice with MOE produced a significant reduction (*P* ≤ 0.05) in the WBC count to a level near to that of the control.

**Table 3:** Effects of *Moringa* seed oil extract on hematological parameters of EAC mice

**Groups**	Duration	C	MOE	EAC	EAC/ MOE
**Parameters**					
RBCs × (10^6^/µl)	Week 1	9.74 ± 0.13^a^	9.40 ± 0.30^a^	7.40 ± 0.05^c^	8.57 ± 0.99^b^
	Week 2	9.53 ± 0.29^a^	9.76 ± 0.13^a^	7.10 ± 0.05^c^	8.33 ± 0.20^b^
Hb (g/dl)	Week 1	14.56 ± 0.23^a^	14.06 ± 0.12^a^	7.43 ± 0.29^c^	10.90 ± 0.20^b^
	Week 2	14.66 ± 0.33^a^	14.76 ± 0.16^a^	5.30 ± 0.57^b^	9.00 ± 0.00^c^
PCV (%)	Week 1	50.00 ± 1.15^a^	48.53 ± 0.74^a^	31.16 ± 0.16^c^	40.76 ± 0.76^b^
	Week 2	48.33 ± 0.88^a^	50.00 ± 1.15^a^	26.00 ± 0.57^c^	38.00 ± 0.57^b^
MCV (fl)	Week 1	51.31 ± 0.92^a^	52.76 ± 2.18^a^	42.11 ± 0.17^c^	47.54 ± 0.35^b^
	Week 2	50.80 ± 1.93^a^	51.18 ± 0.66^a^	36.61 ± 0.51^c^	45.63 ± 1.00^b^
MCH (pg)	Week 1	14.94 ± 0.02^a^	15.00 ± 0.58^a^	10.04 ± 0.32^c^	12.70 ± 0.37^b^
	Week 2	15.39 ± 0.19^a^	15.12 ± 0.38^a^	7.46 ± 0.07^c^	10.81 ± 0.26^b^
MCHC (%)	Week 1	29.15 ± 0.54^a^	28.99 ± 0.23^a^	23.48 ± 0.82^c^	26.77 ± 0.98^b^
	Week 2	30.36 ± 0.81^a^	29.57 ± 0.99^a^	20.39 ± 0.38^c^	23.69 ± 0.36^b^
WBC × (10^3^/µl)	Week 1	7.00 ± 0.11^c^	7.10 ± 0.12^c^	12.40 ± 0.05^a^	7.80 ± 0.08^b^
	Week 2	6.70 ± 0.14^b^	6.70 ± 0.14^b^	11.60 ± 0.05^a^	6.60 ± 0.20^b^

Values are presented as means (*n* = 14 mice/group) ± SE; different letter superscripts in the same row considered significant at (*P* ≤ 0.05)

*C* Control, *MOE Moringa* seed oil extract, *EAC* Ehrlich ascites carcinoma, *EAC/MOE* Ehrlich ascites carcinoma treated with *Moringa olifera* oil extract

### Serum biochemistry

The biochemical parameters at weeks 1 and 2 are illustrated in Table 4. The biochemical parameters (TC, ALT, AST, ALP, creatinine and urea) at week one did not vary (*P* < 0.05) among the MOE mice and the normal control ones. However, the latter values exhibited a significant increase (*P* ≤ 0.05) in the EAC mice when compared to the control ones. Treatment of EAC mice with MOE reduced (*P* ≤ 0.05) cholesterol, AST, ALT, ALP, creatinine and urea levels as compared to the non-treated EAC group. Other biochemical parameters (glucose, TP, globulin, albumin and A/G ratio) were not significantly varied (*P* 0.05) among the MOE control and the normal control groups. However, the values were significantly lesser (*P* ≤ 0.05) in the EAC mice than the control ones. Treatment of EAC mice with MOE promoted (*P* ≤ 0.05) their values when compared with the untreated EAC group. At week 2, the biochemical parameters (TC, AST, ALT, ALP, creatinine and urea) were not significantly different (*P* > 0.05) in the control MOE mice and the control mice, while AST and ALT activities increased (*P* ≤ 0.05) when came in comparison to the control mice. However, the former parameters were significantly higher (*P* ≤ 0.05) in the EAC group than in control. Treatment of EAC mice with MOE significantly reduced (*P* ≤ 0.05) TC, AST, ALT, ALP, creatinine and urea when compared to EAC mice that received no treatment. Concerning glucose, TP, globulin, albumin and A/G ratio, non-significant change (*P* > 0.05) among the control MOE group and the control group. However, glucose, TP, globulin, albumin and A/G ratio values were reduced (*P* ≤ 0.05) significantly in the EAC mice when compared with the control ones. MOE gavage to EAC mice promoted (*P* ≤ 0.05) the later parameters compared to the EAC untreated mice. MOE promoted (*P* ≤ 0.05) the values of glucose, TP, urea and the A/G ratio near the normal control.

**Table 4:** Effects of *Moringa* seed oil extract on serum biochemical parameters of EAC mice

**Groups**	Duration	C	MOE	EAC	EAC/MOE
**Parameters**					
Cholesterol (mg/dl)	Week 1	142.30 ± 1.40^c^	140.30 ± 1.40^c^	163.00 ± 1.70^a^	151.00 ± 1.00^b^
	Week 2	122.60 ± 1.45^c^	112.30 ± 1.45^d^	178.30 ± 0.88^a^	128.00 ± 1.52^b^
ALT (U/L)	Week 1	76.30 ± 1.85^c^	77.60 ± 2.18^c^	95.60 ± 1.76^a^	85.30 ± 1.45^b^
	Week 2	67.66 ± 1.45^c^	69.00 ± 1.52^c^	117.60 ± 1.45^a^	103.00 ± 2.08^b^
AST (U/L)	Week 1	118.30 ± 2.02^c^	122.00 ± 1.45^c^	250.00 ± 11.50^a^	187.00 ± 4.33^b^
	Week 2	97.66 ± 1.45^c^	99.33 ± 1.45^c^	129.33 ± 0.66^a^	125.33 ± 0.33^b^
ALP (U/L)	Week 1	118.30 ± 0.88^c^	116.00 ± 1.0^c^	158.00 ± 4.61^a^	136.30 ± 2.02^b^
	Week 2	129.60 ± 1.45^c^	131.00 ± 1.52^c^	147.30 ± 1.76^a^	130.60 ± 0.66^c^
Creatinine (mg/dl)	Week 1	0.24 ± 0.003^c^	0.24 ± 0.003^c^	0.32 ± 0.01^a^	0.28 ± 0.01^b^
	Week 2	0.20 ± 0.00^d^	0.14 ± 0.02^e^	0.50 ± 0.01^a^	0.24 ± 0.01^bc^
Urea (mg/dl)	Week 1	57.00 ± 2.6^ cd^	54.30 ± 2.96^d^	74.00 ± 0.57^a^	63.00 ± 1.73^bc^
	Week 2	41.30 ± 3.5^b^	30.00 ± 0.00^c^	59.30 ± 0.66^a^	44.00 ± 3.0^b^
Glucose (mg/dl)	Week 1	112.00 ± 1.15^a^	110.00 ± 1.15^a^	74.60 ± 0.88^d^	87.30 ± 6.35^c^
	Week 2	97.66 ± 1.45^a^	97.66 ± 1.45^a^	67.00 ± 1.52^d^	81.66 ± 5.84^c^
T. protein (g/dl)	Week 1	5.90 ± 0.02^a^	6.30 ± 0.05^a^	5.20 ± 0.14^b^	5.90 ± 0.14^a^
	Week 2	5.80 ± 0.08^a^	5.60 ± 0.11^ab^	4.30 ± 0.14^c^	5.50 ± 0.03^ab^
Albumin (g/dl)	Week 1	3.40 ± 0.08^a^	3.30 ± 0.05^a^	2.40 ± 0.08^c^	2.80 ± 0.03^b^
	Week 2	2.80 ± 0.03^a^	2.70 ± 0.03^ab^	1.90 ± 0.06^c^	2.60 ± 0.06^b^
Globulin (g/dl)	Week 1	2.40 ± 0.08^c^	2.50 ± 0.06^c^	3.20 ± 0.14^a^	2.80 ± 0.02^b^
	Week 2	2.90 ± 0.08^a^	2.80 ± 0.08^ab^	1.90 ± 0.03^c^	2.70 ± 0.03^ab^
A/G ratio	Week 1	1.80 ± 0.05^a^	1.80 ± 0.05^a^	1.00 ± 0.00^c^	1.40 ± 0.03^ab^
	Week 2	1.06 ± 0.07^a^	1.00 ± 0.04^a^	0.68 ± 0.01^c^	0.95 ± 0.01^ab^

Values are presented as means (*n* = 14 mice/group) ± SE; different letter superscripts in the same row considered significant at (*P* ≤ 0.05). C: Control, MOE: *Moringa* seed oil extract, EAC: Ehrlich ascites carcinoma, EAC/MOE: Ehrlich ascites carcinoma treated with *Moringa olifera* oil extract

### Inflammatory markers

The levels of IL-2, IL-6 and TNFα exhibited non-significant (*P* > 0.05) variations in the control MOE mice when matched to the control ones at weeks 1 and 2 of the experimental period. However, their levels increased (*P* ≤ 0.05) significantly in the EAC mice when compared with the control ones at weeks 1 and 2. MOE administered to EAC mice significantly (*P* ≤ 0.05) reduced IL-2, TNFα and IL-6 levels in comparison to the EAC non-treated group at weeks 1 and 2 (Table 5).

**Table 5:** Effects of *Moringa* seed oil extract on serum cytokines of EAC mice

Groups	Duration	C	MOE	EAC	EAC /MOE
Parameters					
IL-2 (pg/mL)	Week 1	26.80 ± 0.06^d^	26.80 ± 0.08^d^	46.50 ± 0.39^a^	35.40 ± 0.27^c^
	Week 2	27.00 ± 0.07^d^	26.70 ± 0.06^d^	57.50 ± 0.37^a^	29.10 ± 0.23^c^
IL-6 (pg/mL)	Week 1	4.50 ± 0.03^d^	4.50 ± 0.02^d^	11.00 ± 0.19^a^	8.20 ± 0.11^c^
	Week 2	4.60 ± 0.03^d^	4.50 ± 0.01^d^	15.00 ± 0.2^a^	6.50 ± 0.11^c^
TNFα (pg/mL)	Week 1	32.20 ± 0.14^d^	31.80 ± 0.07^d^	69.70 ± 0.49^a^	52.20 ± 0.25^c^
	Week 2	32.10 ± 0.14^d^	31.70 ± 0.05^d^	94.00 ± 0.66^a^	40.30 ± 0.44^c^

Values are presented as means (*n* = 14 mice/group) ± SE; different letter superscripts in the same row considered significant at (*P* ≤ 0.05)

*C* Control, *MOE* *Moringa* seed oil extract, *EAC* Ehrlich ascites carcinoma, *EAC/MOE* Ehrlich ascites carcinoma treated with *Moringa olifera* oil extract

### Lipid peroxidation and antioxidants

Hepatic GSH, SOD and MDA values in the MOE group were not significantly altered (*P* > 0.05) when matched with that in the control one at weeks 1 and 2 of the experimental period. However, the EAC group exhibited a significantly (*P* ≥ 0.05) decreased GSH and SOD when matched to the control one, while elevated MDA levels were observed at weeks 1 and 2. Administration of MOE to EAC mice significantly (*P* ≤ 0 0.05) promoted GSH and SOD activity when matched to the EAC group while ameliorating (*P* ≥ 0.05) the elevated MDA at week 1 and week 2 of the experimental period. Renal GSH, SOD and MDA echelons in the MOE group were not significantly altered (*P* > 0.05) when matched with that in the control group at weeks 1 and 2 of the experimental period. However, the EAC group exhibited a significant decrease (*P* ≥ 0.05) in SOD activity and GSH compared to the control group, while elevated MDA levels were observed at weeks 1 and 2. Administration of MOE to EAC mice significantly (*P* ≥ 0.05) promoted GSH and SOD activity when matched to the EAC group while ameliorating (*P* ≥ 0.05) the elevated MDA at weeks 1 and 2 of the experimental period (Table 6).

**Table 6:** Effects of *Moringa* seed oil extract on antioxidants of liver and kidney tissues of EAC mice

Groups	Organ	Duration	C	MOE	EAC	EAC/MOE
**Parameters**						
GSH (mg/g)	Liver	Week 1	11.23 ± 0.01^a^	11.57 ± 0.03^a^	7.68 ± 0.03^c^	9.75 ± 0.01^b^
		Week 2	11.28 ± 0.03^a^	11.68 ± 0.03^a^	7.78 ± 0.03^c^	9.80 ± 0.04^b^
	Kidney	Week 1	6.52 ± 0.02^a^	6.62 ± 0.01^a^	4.21 ± 0.06^c^	5.74 ± 0.01^b^
		Week 2	6.62 ± 0.20^a^	6.72 ± 0.01^a^	4.27 ± 0.09^d^	5.79 ± 0.40^b^
SOD (U/g)	Liver	Week 1	5.65 ± 0.01^a^	5.81 ± 0.01^a^	3.11 ± 0.03^c^	5.11 ± 0.03^b^
		Week 2	5.78 ± 0.03^b^	5.91 ± 0.01^ab^	3.12 ± 0.04^d^	5.11 ± 0.04^c^
	Kidney	Week 1	3.59 ± 0.01^a^	3.63 ± 0.01^a^	1.77 ± 0.00^c^	2.85 ± 0.04^b^
		Week 2	3.69 ± 0.00^a^	3.73 ± 0.00^a^	1.87 ± 0.00^d^	2.90 ± 0.00^b^
MDA (nmol/g)	Liver	Week 1	0.23 ± 0.03^c^	0.22 ± 0.02^c^	0.48 ± 0.00^a^	0.33 ± 0.03^b^
		Week 2	0.29 ± 0.00^c^	0.27 ± 0.00^c^	0.59 ± 0.00^a^	0.37 ± 0.00^b^
	Kidney	Week 1	0.11 ± 0.00^c^	0.12 ± 0.38^c^	0.30 ± 0.00^a^	0.19 ± 0.00^b^
		Week 2	0.12 ± 0.00^c^	0.13 ± 0.00^c^	0.55 ± 0.00^a^	0.33 ± 0.00^b^

Values are presented as means (*n* = 14 mice/group) ± SE; different letter superscripts in the same row considered significant at (*P* ≤ 0.05)

*C* Control, *MOE* *Moringa* seed oil extract, *EAC* Ehrlich ascites carcinoma, *EAC/MOE* Ehrlich ascites carcinoma treated with *Moringa olifera* oil extract

### Histopathological results

**Fig. 1:**
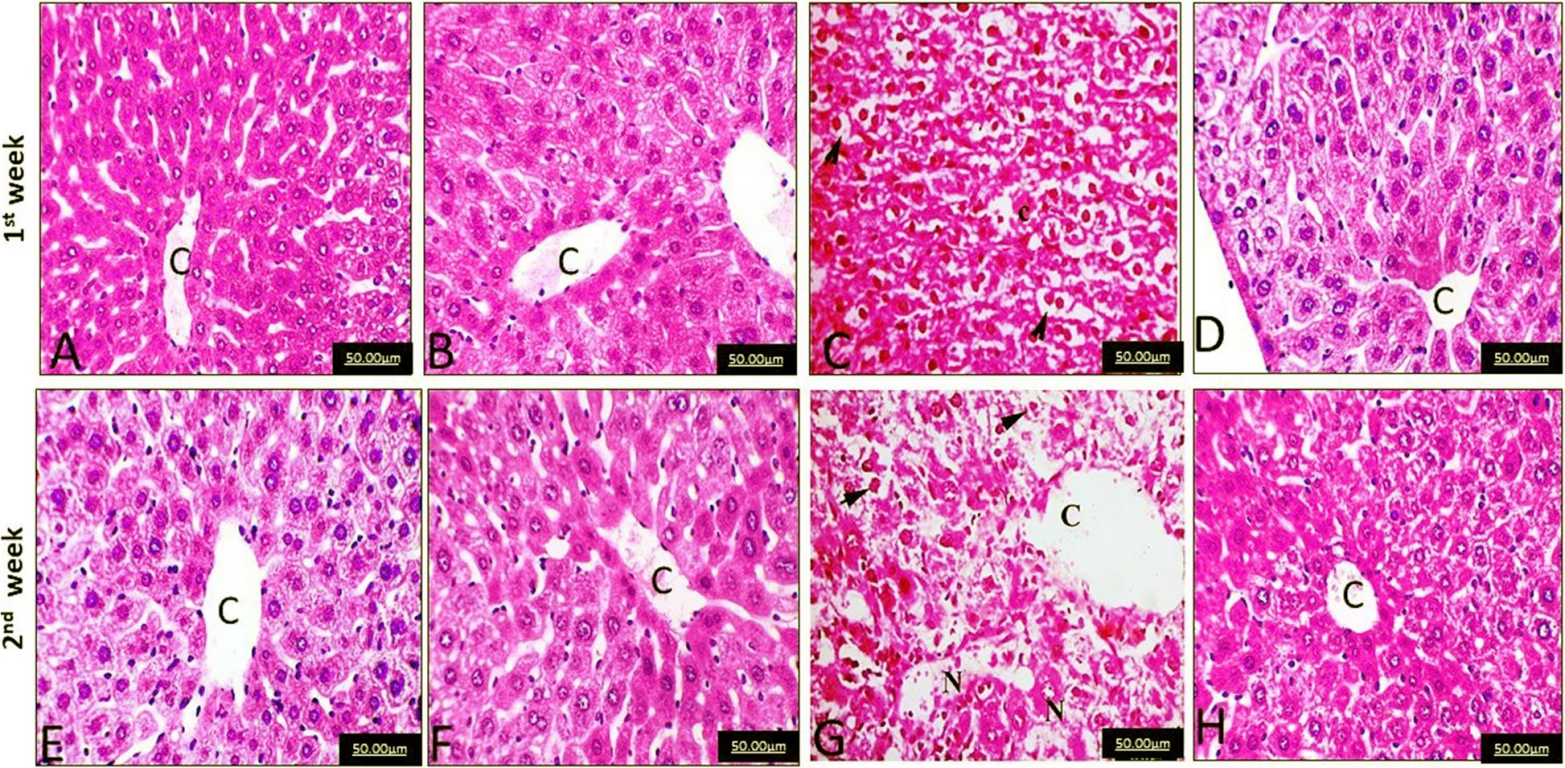
**A** and **E** Normal hepatic lobules, central vein (C), and hepatic cells in the livers of the control group after 1 and 2 weeks of treatment. **B** and **F** Normal hepatic lobules, central vein (C), and hepatic cells in the liver of the *Moringa* seed oil extract (MOE) group after 1 and 2 weeks of treatment. **C** and **G** Severe congestion of hepatic blood vessels, diffuse vacuolar degeneration (arrow), and necrotic nuclei of hepatic cells (N) in the Ehrlich ascites carcinoma (EAC) group after 1 and 2 weeks of treatment. **D** and **H** Normal hepatic lobules, central vein (C), and hepatic cells in the liver of the EAC group treated with MOE after 1 and 2 weeks of treatment (H&E, × 400)

After the first and second weeks, the livers of the control groups displayed normal hepatic lobules, central veins and hepatic cells (Fig. 1A and E). Hepatic cells are hexagonal with centrally located nuclei and plentiful eosinophilic cytoplasm. The same histological structure as the control group was observed in the MOE group after the first and second weeks (Fig. 1B and F). On the contrary, livers of mice injected with EAC cells after the first and second weeks revealed multiple congestion of central veins along with diffuse vacuolar degeneration of hepatic cells and necrotic nuclei of hepatic cells (Fig. 1 C &G). After the first and second weeks, the EAC/MOE group showed normal hepatic lobules with radiating cords of hepatic cells surrounding the central vein (Fig. 1 D &H).

**Fig. 2:**
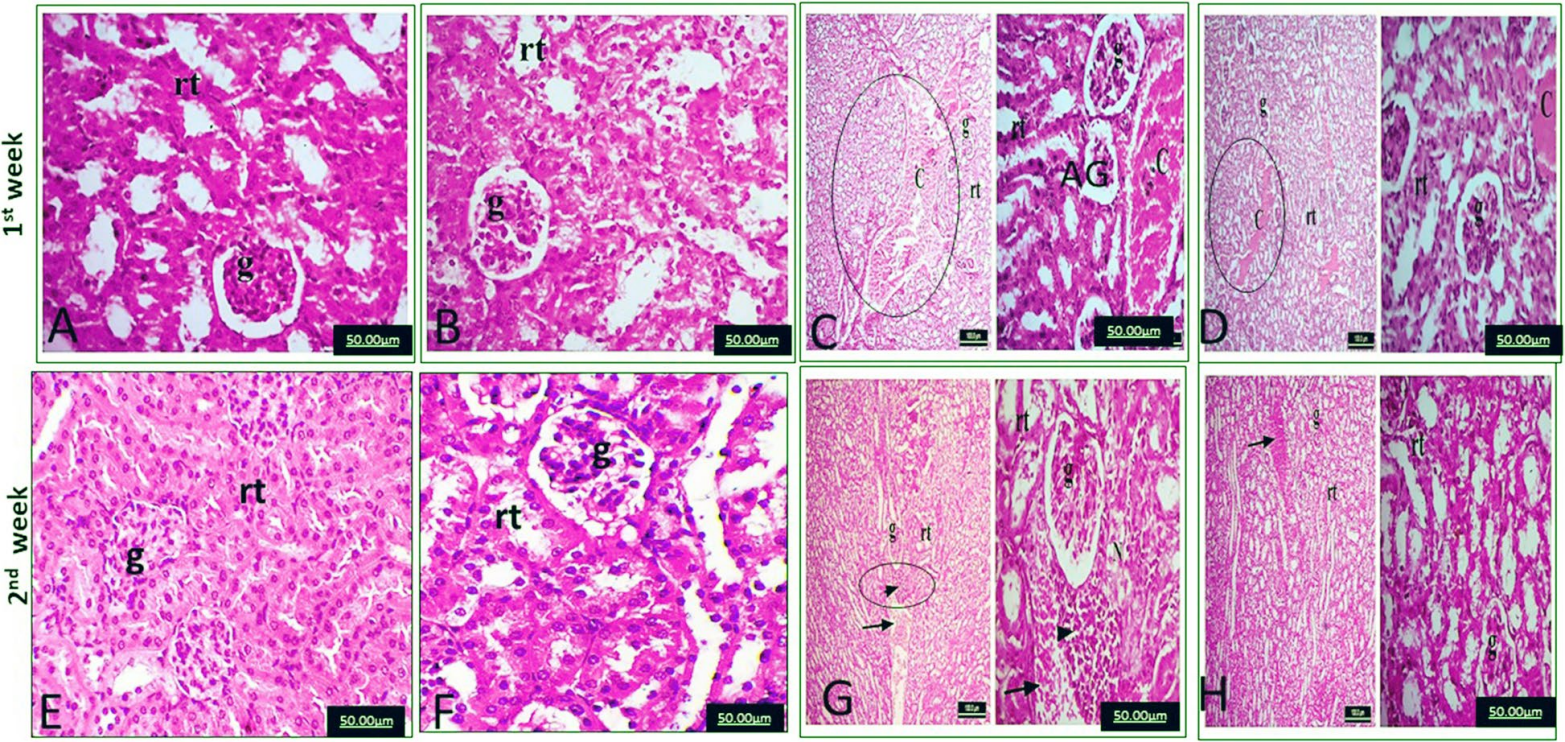
**A** and **E** Normal glomeruli (g) and renal tubules (rt) in the kidney of the control group after 1 and 2 weeks of treatment. **B** and **F** Normal glomeruli (g) and renal tubules (rt) in the kidneys of the *Moringa* seed oil extract (MOE) group after 1 and 2 weeks of treatment. **C** Severe congestion of renal blood vessels (c) and atrophic glomerulus (AG) in the kidney of the Ehrlich ascites carcinoma (EAC) group after 1 week of treatment. **G** Severe congestion of renal blood vessels (c) and periglomerular lymphocytic infiltration (arrowhead) in the kidneys of the EAC group after 2 weeks of treatment. **D** and **H** Normal glomeruli (g) and renal tubules (rt), in addition to mild congestion of the renal tubules to the kidney of the EAC group treated with MOE after 1 and 2 weeks of treatment (H&E, × 400)

The kidneys of the control and MOE groups after the first and second weeks showed normal tissue histoarchitecture of both proximal and distal convoluted tubules as well as glomeruli (Fig. 2 A &E; Fig. 2 B &F), respectively. After the first week, the kidneys of the EAC group showed severe congestion of the renal blood vessels and atrophic glomeruli (Fig. 2C). After the second week, the kidneys of the EAC group showed severe congestion of the renal blood vessels and focal periglomerular lymphocytic infiltrations (Fig. 2G). After the first and second weeks, the kidneys of the EAC/MOE group showed normal glomeruli and renal tubules, besides mild congestion of the renal tubular blood vessels (Fig. 2 D & H).

### HPLC analysis of MOE extract

HPLC analysis for the MOE extract identified Gallic acid, Cinnamic acid, Ellagic acid, Quercetin, Vanillin, and Hesperidin as major compounds with concentrations of 5.19, 0.48, 3.53, 1.73, 0.53, 1.11 µg/mL as seen in Table 7 supplementary (1).

**Table 7:** Identified compound inside MOE using HPLC

MOE		
Compound	**Area**	**Conc. (µg/ml)**
Gallic acid	13.18	5.19
Chlorogenic acid	0.00	0.00
Catechin	0.00	0.00
Methyl gallate	0.00	0.00
Coffeic acid	0.00	0.00
Syringic acid	0.66	0.28
Pyro catechol	1.23	0.89
Rutin	0.00	0.00
Ellagic acid	2.08	3.53
Coumaric acid	0.00	0.00
Vanillin	3.06	0.53
Ferulic acid	0.00	0.00
Naringenin	0.00	0.00
Daidzein	2.05	0.65
Querectin	2.87	1.73
Cinnamic acid	4.77	0.48
Apigenin	0.00	0.00
Kaempferol	0.00	0.00
Hesperetin	4.28	1.11

### Molecular docking towards GSH and SOD proteins

**Fig. 3:**
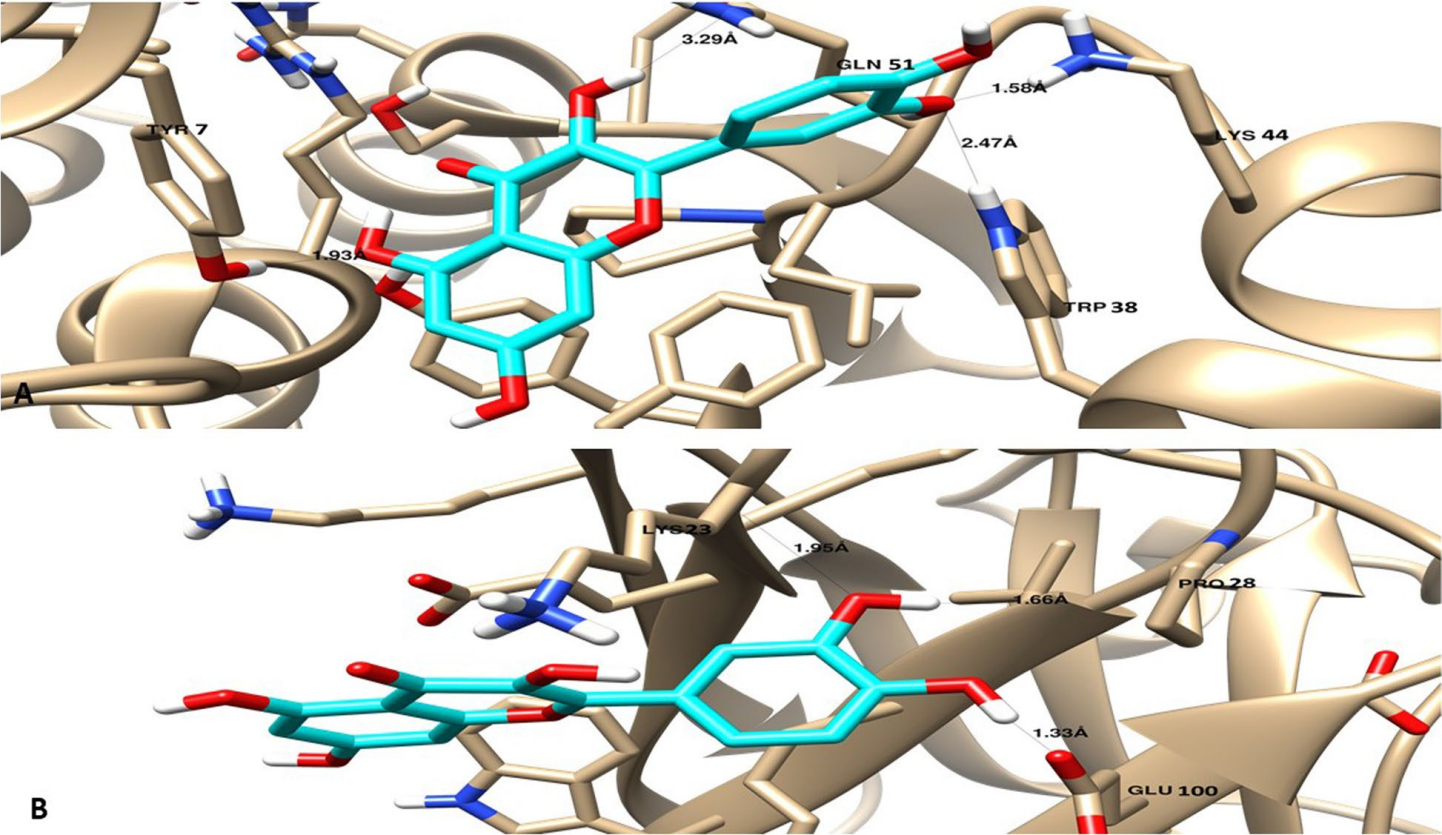
Binding disposition and ligand-receptor interactions of the docked compound Quercetin towards the GSH **A** and SOD **B** proteins. Docking was carried out using AutoDock vina and visualized by Chimera-UCSF

The molecular targeting for the antioxidant activity of MOE extract binding mode of the identified compounds towards the GSH and SOD proteins were tested using the molecular docking study. Most of the major identified compounds exhibited promising binding affinities with good interactions with the key amino acids like the co-crystallized ligand, especially Quercetin with a binding energy of -1669 kcal/mol against GSH protein and –13.97 kcal/mol against SOD proteins. As seen in Table 8, ligand-receptor interactions of the docked compounds were summarized with binding energies. As seen in Fig. 3, Quercetin maintained the binding mode like the co-crystallized ligand and made stable four H-bonds with Lys 44, Trp 38, Gln 51 and Tyr 7 and it formed Indued-induced dipole interactions with Phe 8 inside the GSH protein, while it formed three H-bonds with Lys 23, Pro 28 and Glu 100 inside the SOD protein. Accordingly, docking studies may highlight the proposed binding mode for the most active compound with antioxidant activity and future studies will be continued to isolate and investigate the detailed molecular target and mechanism of Quercetin.

**Table 8:** Summary of ligand-receptor interactions of the identified major compounds towards the GSH and SOD proteins

Compound	GHS (PDB = 2A2R)		SOD (PDB = 4A7U)	
	**Binding energy (Kcal/mol)**	**Ligand-receptor interactions**	**Binding energy (Kcal/mol)**	**Ligand-receptor interactions**
Co-crystallized ligand	-11.3	6 H-bonds Ser 65, Leu 52, Lys 44, Gln 64, Trp 38 and Gln 51	-10.6	3 H-bonds with Lys 23, Pro 28 and Glu 100
Cinnamic acid	-12.19	1 H-bond with Ser 65 Ion–dipole interaction with Arg 13	-8.76	2 H-bonds with lys 23 and Pro 28 Ion–dipole interaction with Lys 23
Ellagic acid	-17.9	2 H-bonds with Leu 52 and Lys 44	-8.52	3 H-bonds with Pro 28, Lys 23 and Glu 100
Quercetin	-16.69	4 H-bonds with Lys 44, Trp 38, Gln 51, and Tyr 7, Indued-induced dipole force with Phe 8	-13.97	3 H-bonds with Lys 23, Pro 28 and Glu 100
Gallic acid	-16.45	2 H-bond with Gln 51 and Leu 52	-9.7	4 H-bons with Lys 23, Pro 28, Glu 100
Vanillin	-12.98	1 H-bond with Ser65. Ion–dipole with Arg 13	-8.2	2 H-bonds with Lys 23 and Pro 28
Hesperidin	-11.98	2 H-bonds with Lys 44, Trp 38. Indued-induced dipole force with Phe 8	-9.2	2 H-bonds with Lys 23, and Glu 100

## Discussion

Cancer is a global problem that causes seriously compromises health, leading to death and adverse economic consequences [35]. Medicinal plants have recently been introduced as anticancer therapeutic agents because of their high safety and fewer side effects in use [36]. *Moringa oleifera* is a medicinal plant with several favorable therapeutic effects [24]. The present study investigated the in vivo anticancer and antioxidant potential of MOE in female EAC mice, with special emphasis on identified compounds using HPLC.

A previous report showed that Gallic acid, the major compound found in MOE, has antitumor effects [37]. Therefore, the richness of MOE with antitumor compounds was confirmed.

The increase in the body weight EAC mice that was observed in the current study agrees with that of Gupta et al*.,* [38]. This increase in body weight could be ascribed to the accumulation of ascitic fluid in the abdominal cavity [39]. Administration of MOE to EAC mice significantly reduced the ascitic fluid-induced body weight increase that was consistent with the lower tumor cell viability in EAC/MOE mice. Barhoi et al*.* [40]*,* reported similar results in mice with MOE, which can inhibit EAC cell growth by inducing apoptosis through the regulation of BAK and nuclear factor kappa B gene expressions [41].

The alteration of hematological parameters in EAC mice represented a reduction in RBC count, Hb concentration, PCV% and blood indices that resulted in microcytic hypochromic anemia. Anemia occurs primarily because of a reduction in erythrocytes number or Hb production, which may ensue either due to hemolytic conditions or iron deficiency [42]. Anemia has been commonly observed in ascites carcinoma [43], whereas EAC cells may lead to hemolysis [44]. Therefore, anemia may occur because of iron deficiency in myelopathic or hemolytic conditions [45]. Another explanation could be related to the downregulation of erythropoietin, which is mediated by pro-inflammatory cytokines that causes iron metabolism deficiency [46]. The overproduction of TNFα in EAC mice inhibited Hb production in this group. Decreased serum iron, erythropoiesis and erythrocytes’ survival have been observed in patients suffering from chronic inflammatory diseases, which may be due to increased TNFα [47]. Moreover, erythrocytes’ membranes are prone to oxidative damage because of their high polyunsaturated fatty acid content [48]. Erythrocytes are also susceptible to peroxide stress due to the unceasing challenge with high oxygen tension, as well as their high iron content, which is considered a strong catalyst for reactive oxygen species (ROS) production [49].

The higher WBC count in EAC mice was in harmony with those reported by Bala et al*.*, [50]. The increased WBC count may be due to tumor development or carcinogenesis, which generates highly diffusible and toxic ROS. The latter causes widespread cellular adducts or damage to biomolecules that cause malignant transformation [51]. In addition, the promotion of TNF-α in EAC mice severely influences the migration and adhesion capabilities of WBC and regulates macrophage activation [52]. Moreover, it regulates blood cell production and lymphocytes development that could explain the increase in WBC count in the EAC group, as well as the observed anemia [53]. The amelioration of the decreased RBC and Hb levels while reducing the elevated WBC in EAC/MOE mice is coincided with that reported by Abd Eldaim et al*.*, [54]. Administration of MOE had a positive effect on the RBC count, Hb content and WBC count, which were close to the normal ranges after the treatment. These signify that MOE has a protective influence on the hematopoietic system, which may be due to its anti-inflammatory and antioxidant potentials [55]. Whereas, the antioxidant constituents of MOE included Gallic acid, Syringic acid, Pyro catechol, Ellagic acid, Vanillin, Daidzein, Quercetin and Cinnamic acid as demonstrated by HPLC in the present study.

Hepatotoxicity has been induced by diverse carcinogens in several animal models [56]. The observed increase in ALT, ALP and AST serum activities may be interpreted as hepatic damage or membrane permeability alteration [57], which suggested hepatocellular damage induced by EAC [58]. This was augmented by the observed histopathological retrogressive changes in the liver of the EAC group. The increase in TC levels may be related to the altered lipid metabolism associated with excess lipogenesis, leading to the pathogenesis of malignancies [59]. Gupta et al*.*, [60] suggested that the increase in TC could be attributed to unsaturated fatty acid peroxidation by free radicals in patients with breast cancer. Administration of MOE to EAC mice ameliorated the increase in TC, AST, ALT and ALP levels that coincide with those of Barhoi et al*.*, [40]. The later reductions could be a result of the variety of antioxidant constituents in MOE, as demonstrated by HPLC analysis, which improve the cellular membrane integrity of the hepatic cells and has hypolipidemic activity [61].

The induced hypoglycemia in EAC-untreated mice may be a result of glucose utilization by tumor cells that overproduce partially processed insulin-like growth factor-2 (IGF-2), which stimulates insulin receptors [62]. The reduction in albumin levels in EAC-induced mice could be due to the presence of hepatic damage caused by cancer cells invasion [63], which manifested as abridged biosynthetic abilities [64]. The former was augmented by the hepatic histopathological results. The observed amelioration of hypoglycemia by MOE in EAC mice may be attributed to the reduction in IGF2 production, [65]. In addition, the antioxidant effect of the MOE active ingredients may be reasonable to the maintained hepatocyte integrity and the increase of their ability to synthesize proteins that was supported by the hepatic histopathological results.

The increase in creatinine and urea levels in the EAC mice is in harmony with the results of Mutar et al*.*, [66], which could be attributed to renal dysfunction [67]. Moreover, the catabolic effect of tumors that increase urea production [68]. The kidney toxicity that accompanies tumorigenesis may result from oxidative damage that was the eventual outcome of excessive ROS generation as well as cytokines [69], as noted in the present study. Oxidative damage, manifested as increased lipid peroxidation and a reduction in GSH content, SOD and catalase activities, leads to renal dysfunction [70]. Furthermore, kidney injury was clear in the histopathological section of the present study, which may result in the descent of albumin in urine [71]. Administration of MOE to EAC mice significantly ameliorated this increase in urea and creatinine levels. The nephroprotective activity of MOE may be attributed to its antioxidant and free radical scavenging abilities [72], which maintain the integrity of the kidney and its function in reducing creatinine and urea as well as histopathological lesions.

The increase in liver and kidney MDA of the EAC group was in agreement with those reported by Medhat et al*.*, [73]. On the other hand, GSH and SOD levels were low in EAC group. The increase in MDA levels could be attributed to the ROS that was generated in the cancer tissues, resulting in lipid peroxidation and subsequent promotion in MDA content, as well as other thiobarbituric acid reactants that cause cellular macromolecule degradation [74]. Moreover, an increase in lipid peroxidation in EAC tumor mice may be closely linked to the observed increase in cholesterol in this group, as mentioned by Ghosh et al*.*, [75]. Parallel, the decrease in GSH and SOD levels might be attributed to the reduction in glutathione content in erythrocytes of EAC-inoculated mice [76]. The depleted GSH content was found to be related to and accompanied by an increased risk of malignancy, as well as an impaired immune response [77]. It has been found that a reduction of SOD activity in EAC-inoculated mice could be attributed to the loss of mitochondria as well as the loss of Mn^+2^ containing SOD activity in EAC cells, leading to a reduction in hepatic SOD activity [78]. Administration of MOE ameliorated the increase in MDA and the decreased levels of renal and hepatic GSH and SOD. These changes may be due to the antioxidant compounds of MOE as long-established by HPLC analysis in the current study. Nadro et al*.*, [79] found that MOE possesses the ability to inhibit lipid peroxidation.

The upregulation of IL-2 and IL-6 could be attributed to the role of cytokines in immune unresponsiveness and carcinogenesis [80]. Moreover, TNF-α echelons were promoted in the EAC mice that was in harmony with that reported by Gowda et al*.,* [81]. IL-6 is produced by neoplastic or late-phase inflammatory cells, as shown in the EAC group [82]. The production of TNF-α is owing to an increase in macrophage-produced ROS, which promotes lipid peroxidation [83]. MOE administration significantly ameliorated IL-2 and IL-6 levels in EAC-bearing mice that may be mediated either by oxidative stress mitigation-dependent or -independent pathways [84] as well as the anti-inflammatory effects [85] associated with downregulation of the pro-inflammatory genes [86].

Glutathione transferase enzymes (EC 2.5.1.18; GSTs) biotransform several compounds, including some that are carcinogenic, mutagenic, toxic, or therapeutically useful [87]. The antioxidant defense provided by SODs is crucial in the fight against oxidative stress [88]. Previous research has shown that SOD has therapeutic implications and physiological relevance [88]. It has anti-inflammatory properties and protects against malignant transformation of cells [89]. Therefore, molecular docking investigation was utilized to investigate the virtual mechanism of binding of discovered components in MOE extract towards the molecular targets as antioxidant activity GSH and SOD proteins. Docking results as cheminformatics tool exhibited good binding affinities of identified compounds in the MOE extract towards GSH and SOD proteins through their good binding energies and interactive binding modes with the key amino acids for the activity.

## Conclusion

Biological evaluation of EAC toxicity showed disruption in inflammatory cytokines, biochemical parameters, antioxidant enzymes and oxidative stress markers with hepatorenal histopathological changes. Administration of MOE to EAC-bearing mice diminished these disturbances, induced a potent antioxidant effect and normalized most of the tested parameters, as well as hepatorenal histopathology. Therefore, MOE is a promising antitumor candidate that protects the body from oxidation and limits the tumor spread; hence, it can be medically applied in cancer treatment protocols. The molecular docking study highlighted the virtual mechanism of major identified compounds of MOE by HPLC towards GSH and SOD proteins.

## Methods

### Animals

Fifty-six normal female albino mice (22–25 g) were obtained from the laboratory animal house of the Faculty of Veterinary Medicine, Suez Canal University, Egypt. The choice of female mice was according to Vincent and Nicholls [90]. These animals were allowed to acclimatize for two weeks at the laboratory animal house of the Faculty of Veterinary Medicine, Suez Canal University. Mice were set aside at a rate of seven per cage. They were subjected to a natural daylight rhythm at a temperature of 23 ± 2^○^C and were fed a normal basal rodent diet food and water *ad libitum*. Ethical approval was granted for the present study (No. 2021045) by the Faculty of Veterinary Medicine Ethical Committee, Suez Canal University.

### Drugs and chemicals

*Moringa oleifera* seed oil extract (MOE; 100% pure) was purchased from Grenera Nutrients Private Limited, India. It was extracted using the cold press method.

### Induction of EAC in mice

The parent cell line of EAC was obtained from the National Cancer Institute (NCI), Cairo, Egypt. EAC cells were sustained in female Swiss albino mice, in *vivo,* by transplantation of 2.5 × 10^6^ cells/animal via intraperitoneal (IP) route every 10 days [91] under the influence of ketamine-xylazine combination in doses 80 mg/kg and 10 mg/kg, respectively (IP for both). Ascitic fluid was aspirated from EAC tumor-bearing animals at 7^th^ or 8^th^ days post injection under the effect of the later anesthesia. The ascitic fluid was subjected to trypan blue-guided viable cell counting using a hemocytometer [92]. Viable cells were adjusted to 2.5 × 10^6^ viable tumor cells/0.2 mL phosphate-buffered saline.

### Experimental design

After a period of acclimatization that was one week, the experimental animals were assigned into four groups (14 mice/group, randomly) as follows: Group I (Control): Mice were injected with saline in a dose of 0.2 mL/animal by IP route and administered daily corn oil at 1.2 mL/kg orally [93], Group II (MOE): Mice were orally administered MOE (500 mg/kg) daily [94], Group III (EAC): Ehrlich cells-injected mice (2.5 × 10^6^ cells) in a dose of 0.2 mL/animal by IP route that were gavaged daily corn oil (1.2 mL/kg) for two weeks, Group IV (EAC/MOE): EAC-bearing mice were administered oral MOE (500 mg/kg body weight) daily for two weeks. All experimental mice were fed a basal rodent diet with water ad libitum throughout the two-week experimental period. Seven mice per group were euthanized after the first week of treatment. The remaining rats were sacrificed after the second week of treatment. Individual body weights were recorded weekly. Seven mice/ group were euthanized by cervical dislocation under the effect of ketamine-xylazine combination in doses 80 mg/kg and 10 mg/kg, respectively, by IP route after the first week of the treatment [95]. The rest of the groups were euthanized after the second week of the treatment using the same previous procedures.

### Blood sampling

Blood samples were obtained two times, the first time after one week and the second after two weeks (i.e., at the termination of the experiment). Retro-orbital venous blood was drawn from mice fasted for 10 h under the influence of inhalation anesthesia with tetrahydrofuran. The blood was drawn into double tubes; one contained an EDTA anticoagulant. This tube was used for hematological evaluation. The other tube was a plain one that was used for serum separation. The separated sera were used for biochemical and cytokines analysis.

### Hematological parameters evaluation

Hematological parameters were estimated using the standard protocol described by F Imam, NO Al-Harbi, MM Al-Harbi, MA Ansari, KM Zoheir, M Iqbal, MK Anwer, AR Al Hoshani, SM Attia and SF Ahmad [96]. This included erythrocytes count (RBC), packed cell volume (PCV), hemoglobin (Hb) concentration, mean corpuscular hemoglobin concentration (MCHC), mean corpuscular volume (MCV), mean corpuscular hemoglobin (MCH) and total leukocytic (WBC) count. An improved Neubauer hemocytometer and diluting fluids (Haym’s and Turke’s) were used for erythrocytes' and leukocytes' counts, respectively.

### Serum biochemical analysis

Serum biochemical parameters, including cholesterol (TC) Cat. No. STA-384, glucose (Cat. No. STA-680), aspartate aminotransferase (AST) Cat. No. MET-5127, alkaline phosphatase (ALP) Cat. No. CBA-301, alanine aminotransferase (ALT) Cat. No. MET-5123, albumin Cat. No. MET-5017, total protein (TP) Cat. No. CB-P007-K, Creative Biolabs Co., USA were estimated. Creatinine (Cat. No. STA-378, Cell Biolabs, USA) and urea (Cat. No. STA-382, Cell Biolabs, USA) were estimated. Globulin levels were obtained by subtracting albumin from TP and A/G (albumin/globulin) ratio were calculated [97].

### Ascitic fluid sampling and viable tumor cell count

Ascitic fluid was drawn from each experimental mouse model inoculated with EAC cells. These fluids were then subjected to viable tumor cell counts. To obtain the viable tumor cell counts, the peritoneal ascitic fluid was diluted 100 times using trypan blue 0.4% in phosphate buffer saline [98]. The viable cells were counted in 25 secondary squares of the Neubauer chamber, where the non-viable cells responded to the blue stain of trypan blue, while the viable cells did not take the blue color [99]. The number of viable cells in each animal was compared to that of the untreated group. The cell count was determined from the following equation: Cell count = Number of cells × dilution factor/Area × thickness of the liquid film.

### Lipid peroxidation and antioxidants

The liver and kidneys used for preparation of tissue homogenates were removed, washed three times in ice cold saline and blotted individually on ash-free filter paper. Specimens from each organ were separated into two parts. Each piece was weighted and homogenized separately with tissue homogenizer. One part (0.5 g) was homogenized in 5 mL phosphate buffer saline (PBS) 50 mM pH (7.4) for estimation of reduced glutathione (GSH) and superoxide dismutase (SOD). The second (0.5 g) was homogenized in 5 mL potassium phosphate buffer 10 mM pH (7.4) for estimation of malondialdehyde (MDA). The crude tissue homogenate was centrifuged at 10,000 rpm, for 15 min in cold centrifuge and the resultant supernatant was used for the various determination of SOD, MDA and GSH using kits obtained from Bio-Chain (Inc., USA). These markers were estimated according to the manufacturer's instructions.

### Inflammatory markers

The levels of interleukin 2 (IL-2), tumor necrosis factor alpha (TNF-α) and interleukin 6 (IL-6) in the sera were assayed using IBL Co., Japan enzyme-linked immunosorbent assay (ELISA) kits. All measurements were performed in accordance with the manufacturer's enclosed pamphlet instructions.

### Histopathological examination

Formalin-fixed liver and kidney from each experimental animal were subjected to dehydration using ascending ethyl alcohol concentration gradients (70, 80, 90 and 100%) for 1 h each. The specimens were cleared using two changes of xylene (1 h each) and then entrenched in paraffin blocks. Tissue sections (5 μm) were obtained. The microscopic slides were stained with hematoxylin and eosin (H&E), as described by GL Kumar and J Kiernan [100].

### High-Performance Liquid Chromatography (HPLC) analysis of MOE extract

HPLC analysis was performed using an Agilent 1260 series. The Eclipse C18 column (4.6 mm × 250 mm i.d., 5 m) was used for the separation. Water (A) and trifluoroacetic acid in acetonitrile (B) at 0.05% were used as the mobile phase, moving at a rate of 0.9 mL/min. The linear gradient of the mobile phase was as follows: 0% A for 0 min, 80% A for 0–5 min, 60% A for 5–8 min, 70% A for 8–12 min, 82% A for 12–15 min, 82% A for 15–16 min and 82% A for 16–20 min. At 280 nm, the multi-wavelength detector was tested. All sample solutions were injected at a volume of 20 μL. For consistency, a 40 °C temperature was kept in the column.

### Molecular docking

Chimera-UCSF and AutoDock Vina were used for molecular modeling study on Linux-based system. Binding sites inside proteins were identified by measuring the sizes of grid boxes encompassing the co-crystallized ligands after their structures had been generated and optimized in Maestro [101]. The investigated compounds were docked towards the protein structures of GSH (PDB = 2A2R) [102] and SOD (PDB = 4A7U) [103] using AutoDock Vina software following routine work [104]. The results of molecular docking were evaluated by binding activities in terms of binding energy and ligand-receptor interactions. The visualization was then done with Chimera.

### Statistical analysis

SPSS (version 25) for Windows was used to complete the statistical analysis. In this study, one-way analysis of variance (ANOVA) was used to compare all groups. Results are presented as means standard errors (SE). Duncan's multiple range test was used to differentiate between the means. Results were considered significant at a probability level (*P* ≤ 0.05).

### Supplementary Information

**Additional file 1.**

## Data Availability

The authors confirm that the data supporting the findings of this study are available within the article.
