# Supplementary material for: Evaluation of antioxidant, anti-inflammatory, anticancer activities and molecular docking of Moringa oleifera seed oil extract against experimental model of Ehrlich ascites carcinoma in Swiss female albino mice
Source: BMC Complement Med Ther. 2023 Dec 14;23:457. doi: 10.1186/s12906-023-04279-z (PMC10720142; doi:10.1186/s12906-023-04279-z)
Supplement: Supplementary file 1 — Additional file 1. [file 12906_2023_4279_MOESM1_ESM.docx]

**Supplementary 1**


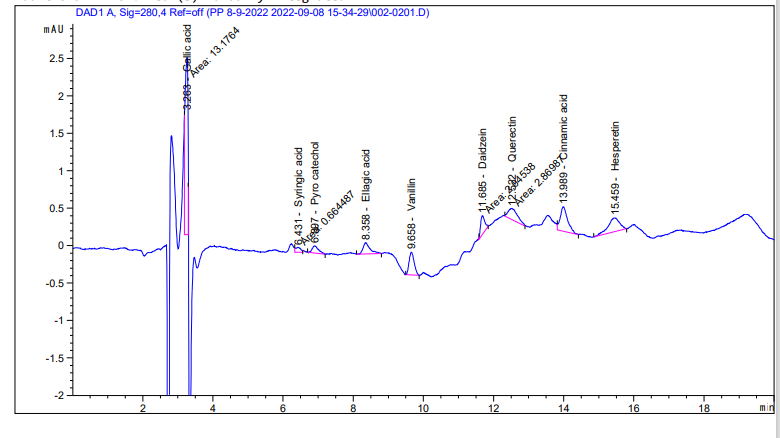


**Representative HPLC chromatogram of moringa oil extract showing Gallic acid, Cinnamic acid, Ellagic acid, Quercetin, Vanillin, and Hesperidin peaks**
